# Supplementary material for: Are carbon emissions trading and green financial instruments synergistic? -Comprehensive quantitative research based on content analysis
Source: PLoS One. 2024 Mar 7;19(3):e0298601. doi: 10.1371/journal.pone.0298601 (PMC10919601; doi:10.1371/journal.pone.0298601)
Supplement: S1 Data — (ZIP) [file pone.0298601.s001.zip › Data/地级市Z_F工作报告.zip!/║╙─╧/Σ≡║╙╩╨/└┤╫╘╣1⁄2╓┌║┼í╛┬φ┐╦╩2╛▌═°í┐_2019.docx]

各位代表：

现在，我代表市人民政府，向大会报告工作，请予审议，并请各位政协委员和其他列席人士提出意见。

一、2018年工作回顾

过去的一年，在省委、省政府和市委领导下，全市上下以习近平新时代中国特色社会主义思想为指导，全面贯彻党的十九大精神和习近平总书记视察指导河南时的重要讲话精神，坚持稳中求进工作总基调，坚持新发展理念引领，坚持高质量发展方向，深入落实新时代漯河经济社会发展“四三二一”工作布局，全面推进“四城同建”，经济社会发展总体平稳、稳中有进、进中向好，在以漯河更加精彩为中原更加出彩增添浓彩中迈出坚实步伐。

一年来，我们着力推进转型升级，发展质量不断提升。统筹推进稳增长、促改革、调结构、惠民生、防风险，经济保持平稳增长。生产总值达到1237亿元，增长7.7 %，高于全省0.1个百分点。规模以上工业增加值增长8.2%，高于全省1个百分点。固定资产投资增长9.6%，高于全省1.5个百分点。社会消费品零售总额增长10.6%，高于全省0.3个百分点。一般公共预算收入88.4亿元、增长6.9%，其中税收占比77.5%，高于全省6.9个百分点。城镇和农村居民人均可支配收入分别增长8%和8.9%，均高于全省0.2个百分点。

积极扩大有效投资。完善重点项目集中开工、观摩讲评机制，建立三大周例会制度，统筹解决项目建设中的各类难题，推动产业转型升级项目和招商引资新上项目、百城提质暨文明创建项目、基础设施补短板项目顺利实施。43个省重点项目完成投资374.4亿元，160个市重点项目完成投资553.5亿元。66个亿元以上项目竣工或主体竣工，94个亿元以上项目有序推进，华润怡宝纯净水等一批项目实现当年开工、当年建成、当年见效。

加快推进工业转型。设立1亿元专项奖补资金，推动实施智能化、绿色化、技术改造“三大改造”项目318个、竣工159个，完成投资218亿元。双汇云商平台投入使用，被评为“河南省智能制造优秀示范企业”。中大恒源成功创建国家级绿色工厂。传统优势产业集中度进一步提高，食品工业增加值增长11.5%，对工业增长贡献率达到69.1%。化工产业增长20.4%。新兴产业增势强劲，高新技术和电子信息产业增加值分别增长26.2%、20.5%。骨干企业群体不断壮大，105家企业纳入全市“十百千”亿级企业及产业集群培育工程，新增超5亿元工业企业10家、超10亿元5家。新增规模以上工业企业76家。临颍产业集聚区、经开区荣获全省优秀产业集聚区。谋划建设食品机械包装、流体科技、电子信息、电力装备四大专业园区，强化产业规划、基础设施建设和龙头企业引进，打造产业集聚区“升级版”。食品机械包装和流体科技产业园成为省装备制造业转型发展示范园区。我市入选全国消费品工业“三品”战略示范城市。

持续壮大第三产业。申通、圆通、顺丰、邮政等区域快递分拨中心，中通、韵达、德邦、百世汇通等快运中心开工建设或竣工投运。双汇物流、大象物流、金顺物流进入中国冷链物流企业百强。快递业务量增长48.9%。全市快递分拨量6.9亿单，在全省仅次于郑州的7.6亿单。电商交易额增长55.5%，邮政业务量增长42.6%。市商务中心区成功晋升一星级。第三产业市场主体数量增长78.4%。服务业投资和税收占全市固定资产投资、税收的比重双双突破50%。服务业占GDP比重达到31.1%，比上年提升2.2个百分点。

提升发展保障能力。探索实施“三块地”改革，土地收储、出让、收益和耕地占补平衡、增减挂钩指标五项工作实现历史性突破。金融机构贷款余额795.4亿元，增长11.5%。通过动产抵押登记和股权、商标权质押帮助219家小微企业融资86.7亿。通过市企业信贷周转资金，为536家（次）企业周转贷款38.6亿元。严格落实减税政策，全口径减免企业税收39亿元。规范PPP运作，在财政部PPP市场透明度报告中，我市居全省第1。建立人才需求定期收集、发布机制，开展职业技能培训5.7万人次。加强电网支撑能力建设，新建、改扩建变电站11座。全社会用电量增长10.1%，其中工业用电量增长8.3%、居全省第6。

一年来，我们着力提升建管品质，城乡面貌不断改观。统筹推进全国文明城市创建、百城建设提质工程暨城市“双修”，全面提高城市规划、建设、管理、经营水平，常住人口城镇化率达到52.47%，在2018年全国文明城市提名城市年度测评中居第33位。

强化规划引领作用。着力加强规划统筹，建立分类、分级决策许可体系，规范市政基础设施规划许可程序，全面启用“规划一张图”网上审批系统。《漯河市城乡规划条例》正式实施。编制完成了城市“双修”和城市设计试点实施方案、城市街道设计导则。审议批准了临颍、舞阳城市总规和中心城区生态水城、“四水同治”、绿地系统等30项专项规划。完善了总体城市设计及部分重点地段城市设计。

完善基础服务功能。全年开工建设中心城区文明创建、百城提质暨城市“双修”项目236个，年度完成投资156亿元，创历史新高。新建、改造牡丹江路、文化路等34条市区道路，打通泰山路、漓江路等10条断头路，牡丹江路沙河桥、中山路上跨立交桥、太白山路沙河桥和澧河桥4座桥梁竣工通车，解放路、太白山路等实现了南环到北环的大贯通。沙颍河复航北舞渡沙河桥、马湾船闸、军李枢纽等控制性工程进场施工。生态水系工程全面铺开，沙澧河大二期高铁以东段、城乡一体化示范区段景观工程和西城区水系工程全面推进；沙澧连通桥闸、丹江路新澧河桥进场施工；幸福渠水系工程107国道以东完成全线征迁；西城区小南湖湿地公园基本完工；经开区中山路循环景观水系建成投用。澧河取水口上移工程开工建设。畅通城市地下“毛细血管”，改造雨污分流管网35公里。铺设供热主管网30公里，新增供热面积98.8万平方米，沙北部分区域首次实现集中供暖。

提升城市管理水平。坚持“颜值”和“内涵”并重，围绕关系群众切身利益的现实问题，全面推进城市“四治”。调整理顺城市管理体制，完善市政设施管理养护、市容市貌标准、城市防汛排涝等操作规范，城市治理逐步纳入规范化、法治化轨道。城市清洁行动持续深化。杨柳絮防治成效显著。汛期内涝问题有效缓解。空中管线乱象逐步规范。17条支路告别无路灯历史。综合整治老旧小区297个、背街小巷599条。新建改造公厕50座、街头游园17个、道路绿化19条、农贸市场6个。依法对1000多处大型违法户外广告进行集中拆除。强降雪期间不间断联合作业，实现了“雪停路通”。渣土清运、公交出租运营等难点问题有效破解，网格化管理、门前“三包”等长效机制不断完善。

实施乡村振兴战略。编制完成乡村振兴战略总体规划。粮食总产181.5万吨，单产447公斤、居全省第3；优质专用小麦、优质大豆、优质花生、小辣椒等优质农产品种植面积持续扩大。新建高标准粮田4.1万亩。主要农作物机械化水平达到88%、居全省第2。新增农民专业合作社等新型经营主体376个。全市流转土地143.3万亩、占家庭承包经营面积的61.5%。农产品加工业与农业总产值之比达到4.5:1，高于全国、全省近一倍。深入开展农村人居环境整治，投入1亿元实施村级公益事业“一事一议”财政奖补项目，投入1675万元推进“厕所革命”，农村保洁市场化运作实现县区全覆盖，农村生活垃圾综合治理通过省级达标验收。完成美丽乡村项目25个。新建改建农村公路135公里。村村通光纤全面提速。在全省首批完成农村承包地确权登记颁证。农村集体资产清产核资大头落地。村“两委”换届顺利完成。

一年来，我们着力推进三大攻坚，短板弱项不断改善。把打好三大攻坚战作为破解瓶颈制约、全面建成小康社会的硬任务，系统谋划、强力推进，实现良好开局。

防范化解重大风险。坚持盘清底数、严控新增，政府存量债务有序化解，隐性债务实现“零增长”。非法集资案件立案数量、涉案金额和参与集资群众数量处于全省较低水平。严密防范食品药品安全风险，食品生产经营日常检查监督全覆盖。非洲猪瘟保持“零疫情”。坚持不间断地排查、整改、消除安全生产隐患，6.6万处隐患完成整改。

深入推进精准脱贫。坚持精准方略，紧盯“两不愁三保障”，扎实推进产业扶贫、就业扶贫、教育扶贫等关键措施，全年投入扶贫资金3.6亿元，资金支出进度居全省第4位；发放扶贫贷款11.3亿元，开工扶贫项目200多个，建成产业、就业基地300多个，带动贫困户8000多户2.6万人；退出贫困村24个，5041人稳定脱贫。探索建立慢性疾病帮扶、精神疾病帮扶、重度残疾康复“三个中心”，有效减少因病致贫、因病返贫的发生。

大力推进污染防治。投入专项资金5亿元，大力实施依法治污、科学治污、精准治污、全民治污，持续开展大气污染、水污染专项整治行动。坚持“日研判调度、周通报、月讲评、季观摩”工作推进机制，聘请千人计划专家，实施大气污染防治精细化管控40条措施。省环保攻坚考核我市水、土两项优秀，大气环境综合指数居全省第5位，综合考核居豫南片区第1名。马沟污水处理厂、召陵污水处理厂二期完工运行，塔河水污染治理、七支渠改造等7项环保工程完工，城区黑臭水体治理全面达标。市区饮用水源地取水水质达标率100%。制定土壤污染防治方案，开展土壤污染状况调查，推进污染治理与修复，土壤环境质量整体保持稳定。

一年来，我们着力深化改革开放创新，动能转换不断加快。坚持改革推动、开放带动、创新驱动，努力为高质量发展带来持久动力。

全面深化改革。“放管服”改革全方位推进。全年累计取消和调整行政职权1523项。全市一体化政务服务平台建成运行。“一网通办、一窗受理、一次办妥、一号对外”全面实施。企业注册登记实施“一次不用跑、一分钱不用掏、三天全办好”运作机制。建设用地预审办理时间由2个月压缩至7个工作日。建设项目手续办理时间压缩1/3以上。不动产登记办理时限压缩至3个工作日以内，当天办结率已达90%。“证照分离”改革走在全省前列。全省首张“零见面”个体工商户营业执照在漯河颁出。跨部门“双随机、一公开”联合监管机制全省推广，市场主体“集群注册”登记全省首创，市场主体活跃度、新登记市场主体和市场主体增速均居全省第一。公共信用信息平台建设全省领先，综合信用指数监测排名跃升至全国第39位、全省第1位。公共资源交易平台实现全流程电子化交易，成为全省首家通过国家认证的三星级平台。机构改革顺利实施，机构设置和职能配置得到优化。“僵尸企业”处置年度任务顺利完成。国有非工企业、盐业体制、事业单位公车、农信社、财税体制等改革扎实推进。

持续扩大开放。积极融入“一带一路”建设，漯河海关获准成立，进口肉类指定口岸功能不断完善，企业主动利用单一窗口报关报检的局面初步形成。全年新签约亿元以上招商引资项目96个。实际利用境外和省外资金增速分别居全省第3和第6。进出口总额增长18.6%。第十六届食博会贸易采购合同总额突破400亿元，增长15.2%。

强力推进创新。实施大中型企业省级研发机构全覆盖、创新龙头企业培育、科技型中小企业培育、创新创业群体培育四大工程，建立创业创新孵化基地奖补机制，49个项目获得科技创新券扶持，21个项目列入全省科技发展计划，财政科技支出占一般公共预算支出的比重达2.54%、全省领先。新建国家级、省级研发平台7家，累计达到60家。全市食品产业公共研发平台和休闲食品协同创新中心、河南省休闲食品工程技术研究中心建成运营。省液压科技产业技术协同创新基地、市液压科技产业技术研究院成功组建。专利授权量1507件，增长18.6%。新增国家级高新技术企业15家，累计达到53家。建成全省第一家食品包装材料省级检测中心和全省唯一的省级童车产品质量监督检验中心。全国首家设立市长标准奖，支持企业主导或参与国家、行业标准制定，我市以标准化建设引领高质量发展的做法全国推广，成为三获国务院大督查表扬的城市。

一年来，我们着力推进共享发展，民生福祉不断增进。全市财政民生支出133.3亿元。省、市民生实事全面完成。累计发放城乡低保补助1.99亿元，4.4万名困难群众生活得到了保障。城乡居民基本养老金最低标准从每人每月82元提高至100元，企业和机关事业单位退休人员基本养老金全部调整到位。城乡居民基本医保财政补助标准、基本公共卫生服务补助标准、困难群众大病保险、城乡医疗救助水平全面提高，“残疾人两项补贴”有效落实。对全市农村和纳入城市低保的适龄妇女免费开展“两癌”筛查，在全市实施预防出生缺陷免费产前筛查和新生儿疾病筛查。建成“职工健康生活馆”50个、“环卫工驿站”31个。着力扩大社会就业，发放创业担保贷款3亿元，扶持农民工返乡创业1.04万人，城镇新增就业4.48万人，新增农村劳动力转移就业2.55万人，城镇登记失业率2.22%。区域性医疗中心建设成效显著，生殖遗传中心、心脑血管中心正式运行，市中心医院获批国家级博士后科研工作站。分级诊疗制度逐步完善。坚持优先发展教育，新建、改扩建幼儿园45所；持续推进“全面改薄”，77所农村薄弱学校改善了办学条件；改扩建26所农村中小学，新增7251个寄宿制学位；普通高考一本、二本上线率分别达到17%和51%；全市11所职业院校全部被评为省级职业教育品牌示范学校或特色学校。高等教育国际化办学实现突破，7个国家的留学生在漯河医专就读。市城市展示馆、图书馆、博物馆、文化馆全面建成开放。第四届许慎文化国际研讨会成功举办。建成村级综合性文化服务中心1117个。承办举办市级以上体育赛事20余项。体育健儿在第13届省运会上取得总分第七、金牌总数第七的历史最好成绩。成功创建河南省公交优先示范城市，新购置260台新能源公交车、新开通线路9条，公交线路实现中心城区全覆盖，“沙澧一卡通”与全国240个城市实现互联互通。新解决7.9万人饮水安全问题。南水北调穿沙工程圆满成功，沙北城区居民全部吃上了丹江水。困难群众法律援助扎实开展，刑事案件法律援助实现全覆盖。深入开展扫黑除恶专项斗争，依法打击各类违法犯罪活动，群众安全感和满意度不断提升。依法做好信访工作，社会大局保持稳定。

各位代表！过去的一年，我们自觉接受人大法律监督、政协民主监督和社会监督，认真办理人大代表建议和政协提案，支持民主党派、工商联和无党派人士参政议政。夯实从严治党主体责任，加强党风廉政建设，严格落实中央八项规定及实施细则精神，推进依法行政，强化审计监督，持续纠“四风”、转作风，政府执行力建设得到加强。精神文明建设成效显著，漯河籍“中国好人”和“感动漯河十大人物”彰显了“崇文尚德、务本图强”的漯河精神。统计、人防、气象、广播电视、史志档案、民族宗教、外事侨务等工作取得新成效，妇女、未成年人、老年人、残疾人等工作进一步加强。驻漯解放军、武警官兵和预备役人员积极支持地方建设，军民融合深入发展，双拥工作持续深化。

回首一年来走过的路，深感成绩来之不易。我们面对的是经济转型阵痛、下行压力加大、多重约束趋紧、改革进入攻坚期的复杂形势，面对的是中美贸易摩擦、非洲猪瘟疫情、一些突发事件的不利影响，面对的是各项攻坚工作越来越多和越来越难、安全稳定矛盾不断增加等繁重任务，实践中我们深刻体会到：第一，必须坚持党的集中统一领导这一根本保证。维护党中央权威和集中统一领导是我国制度优势的根本。一年来，以习近平同志为核心的党中央总揽全局，科学决策，应对复杂形势，保持战略定力，适时预调微调，引领中国经济平稳健康发展。我们在市委的正确领导下，坚决落实党中央国务院决策部署，对中央巡视回头看、中央扫黑除恶督导、中央环保督察和中央、省脱贫攻坚反馈问题逐项整改，举一反三，坚决堵漏洞、补弱项，砂石料厂、“大棚房”等问题得到有效整治；坚决落实党中央稳增长政策措施，着力防下滑、解难题，全市经济实现了平稳健康增长。实践证明，坚持党的集中统一领导既是重大的政治原则，也是做好一切工作的根本保证。我们必须树牢“四个意识”、坚定“四个自信”、坚决做到“两个维护”，确保党中央各项决策部署不折不扣落到实处。第二，必须牢牢把握高质量发展这一根本要求。一年来，我们着眼扩大有效投资，推进产业升级、工业转型，谋划启动了双汇、烟厂、银鸽、际华3515等重点企业五年倍增工程；围绕“规划统筹、项目集成、功能融合”的思路，提前半年谋划论证2019年百城提质暨文明创建项目和近三年项目库；按照“五统一”原则，建立共储、共享的土地收储新机制，着力破解城市发展、项目建设的土地制约；探索推进生态体制改革，实施月度生态补偿、绿色环保调度、污染物排放许可、企业环保信用评价等制度，实现了环境保护与经济社会协同发展。通过一年来的积极探索，全市推动高质量发展的工作思路更加清晰、路径更加明确、机制更加完善。实践证明，高质量发展是经济发展从“有没有”转向“好不好”的发展。我们必须坚定不移走高质量发展之路，持续优化经济结构、转换增长动力，努力实现更高质量、更可持续的发展。第三，必须树牢防患未然这一底线思维。一年来，我们紧盯经济发展、社会稳定、安全生产等重点领域，全面排查，完善机制，着力防范化解重大风险。始终坚持在本级财政可承受范围内，减存量、控增量，确保了政府债务风险总体可控。在省委、省政府领导下，我们按照“四宜四不”原则，依法依规成功处置了非法聚集群体事件。严格落实“六个严禁、六个一律”、“七防五控”、“五控一隔离”等措施，严密防控非洲猪瘟疫情。切实加强问题疫苗药品排查和食品安全监管，大力排查、整改、消除安全生产隐患，守住了不发生系统性区域性风险的底线。实践证明，坚持底线思维、增强忧患意识，是战胜风险挑战、实现稳中求进的重要思想方法、工作方法。我们必须始终保持高度警惕，找准“风险点”，建立“防火墙”，打好防范和抵御风险的有准备之战。第四，必须强化增进民生福祉这一根本目的。一年来，我们着力办好实事保底线，投入财政资金1亿元，打造了两个区域性医疗中心，群众在家门口享受高端医疗服务的梦想正在逐步实现。科学修编中心城区中小学校布局规划，三年内新建和扩建30所中小学校，着力解决城镇中小学“大班额”问题，每个孩子享受公平而有质量的教育的梦想正在逐步实现。坚持尊重历史、实事求是、依法依规化解问题楼盘，16000多户居民拿到了不动产登记证，棚改安置房建成5080套、回迁4168户，完成农村危房改造1170户，老百姓住有所居、居有所安的梦想正在逐步实现。实践证明，民心是最大的政治。我们必须坚持以人民为中心的发展思想，补齐民生短板，让人民群众在共建共享发展中有更多获得感、幸福感。第五，必须保持担当实干这一政治品格。一年来，我们着力强化执行力建设，提振政府工作人员担责、担难、担险的“精气神”，增强担当的意识和责任，营造担当的氛围和机制，提升担当的能力和水平，创新担当的方法和艺术。探索实施重点项目建设、服务企业、城建交通工程三个周例会制度，实现上下级之间及时协调、快速决策，部门之间有效衔接、共同担当，成为以周为工作节点、持续抓落实的重要机制、平台和抓手。全年累计召开周例会131次，研究协调问题573个，解决了一大批时间跨度长、处理难度大的历史遗留问题和当前企业发展、项目建设中的规划、用地、环评、融资、征迁等现实问题。以周例会制度破解难题、推动工作的做法得到了国务院、省委省政府的充分肯定。实践证明，唯有担当，方显忠诚；唯有实干，方有作为。我们必须永葆敢于担当作为的政治品格，久久为功，善作善成，以钉钉子精神做实做细做好各项工作。

过去一年，是辛苦并充实的一年，是付出并收获的一年，这些沉甸甸的收获，是在习近平新时代中国特色社会主义思想指引下，在市委坚强领导下，全市人民攻坚克难、共同拼搏的结果。在此，我代表市人民政府，向工作在各条战线上的工人、农民、知识分子、干部，向驻漯解放军、武警官兵、公安干警、消防救援人员，向所有关心支持漯河发展的各界人士，表示衷心的感谢！

肯定成绩的同时，我们也清醒认识到，面对高质量发展的根本要求，当前还面临不少困难和问题：转型发展任务艰巨。工业中传统产业占比高、高新技术产业占比低、战略性新兴产业还未形成有效支撑，科技创新能力不强，拥有核心技术的创新型企业不多，新生动能替代不够，新技术、新产品、新业态、新模式仍然较少，全社会研究与试验发展经费投入强度不足1%，低于全省、全国平均水平。现代服务业规模较小、占比较低，服务业增加值占生产总值的比重低于全省14.1个百分点、低于全国21个百分点。实体经济比较困难。受多重因素叠加影响，市场主体观望、避险情绪上升，企业投资意愿下降，工业投资增速不高。融资难、融资贵问题尚未得到根本缓解，原材料、土地、人工等因素共同推动企业成本居高不下、盈利能力提升受限，需要我们在加强招商引资的同时，更加注重对现有企业的帮扶和服务，全力推进简政、减税、降费等各项降成本措施的落实。一些领域风险增多。政府债务规模总体较大、风险尚未完全化解；非法集资案件未完全杜绝；食品安全面临较大压力；非洲猪瘟疫情防控仍然面临潜在风险；安全生产和社会稳定领域还存在一些不容忽视的隐患。城市品质有待提升。市区还有240个老旧小区、195条背街小巷需要整治提升，还有超过100公里的管网未实现雨污分流，不少断头路还没有打通，居民集中供热刚刚起步，交通拥堵、停车难问题比较突出，城市公共服务设施相对滞后。民生还有不少短板。城乡居民持续增收制约因素较多，部分群众生活还比较困难，生态环境形势依然严峻，教育、医疗、养老等公共服务还不能很好满足人民群众需要。政府自身建设存在不足。一些干部思想与高质量发展新常态还不适应，政策理解不透、把握不准，专业素养、能力水平有待提高，“四风”问题特别是形式主义、官僚主义新表现不同程度存在，一些领域不正之风和腐败问题时有发生。对此，我们一定要直面问题和挑战，采取更加有力、更加精准的措施，努力把各项工作做到位。

二、2019年工作安排

今年是新中国成立70周年，是全面建成小康社会、实现第一个百年奋斗目标的关键之年。做好政府工作，要认真落实市委七届九次全会要求，以习近平新时代中国特色社会主义思想为指导，全面贯彻党的十九大和十九届二中、三中全会精神，持续落实习近平总书记视察指导河南时的重要讲话和参加十三届全国人大二次会议河南代表团审议时的重要讲话，统筹推进“五位一体”总体布局，协调推进“四个全面”战略布局，坚持稳中求进工作总基调，坚持新发展理念，坚持推动高质量发展，坚持以供给侧结构性改革为主线，坚持深化市场化改革、扩大高水平开放，贯彻“巩固、增强、提升、畅通”的方针，深入开展三大攻坚战，持续推进“四个着力”、打好“四张牌”，深入落实新时代漯河经济社会发展“四三二一”工作布局，全面推进“四城同建”，统筹推进稳增长、促改革、调结构、惠民生、防风险、保稳定工作，保持经济运行在合理区间，进一步稳就业、稳金融、稳外贸、稳外资、稳投资、稳预期，提振市场信心，增强人民群众获得感、幸福感、安全感和满意度，保持经济持续健康发展和社会大局稳定，为全面建成小康社会收官打下决定性基础，在推动漯河更加精彩的征程中迈出更大步伐，为中原更加出彩增添更多浓彩，以优异成绩庆祝中华人民共和国成立70周年。

今年经济社会发展主要预期目标是：经济运行保持平稳，生产总值增长7.5%以上，第一、二、三次产业分别增长3.5%、7%、9%左右，全员劳动生产率增长8%以上，一般公共预算收入增长7%以上，税收占比保持稳定，规模以上工业增加值增长8%，固定资产投资增长8.5%，社会消费品零售总额增长10%以上，居民消费价格涨幅3%左右，消费对经济增长的贡献率进一步提升。创新发展取得新成果，财政科技支出占一般公共预算支出的比重保持全省前列，全社会研究与试验发展经费投入强度力争达到1.25%，科技进步贡献率明显提高，高新技术产业增加值占规模以上工业增加值的比重达到20%。协调发展取得新成效，常住人口城镇化率提高1.5个百分点以上，教育、卫生、文化、体育等社会事业全面进步。绿色发展取得新提升，单位生产总值能耗下降3%，节能减排、环境保护等约束性指标完成省下达任务。开放发展取得新突破，招商引资、实际利用外资和进出口总值稳步增长，营商环境在全省评价排序中进入第一方阵。共享发展取得新进步，居民人均可支配收入增长8%以上，圆满完成省定脱贫目标，城镇新增就业3.4万人以上，城镇调查失业率和城镇登记失业率分别控制在5.5%和4%以内。

落实上述要求和目标，要深入贯彻落实“巩固、增强、提升、畅通”八字方针，着力构建“现代化经济、混合型动力、综合性支撑”三个体系，加快推进“八个高质量发展”：

（一）持续扩大有效投资，增强高质量发展的需求支撑。坚持扩总量与调结构并重，加快实施一批重大项目，扩大投资需求。

加快实施重大项目。围绕产业升级、城乡统筹、基础设施、民生改善等重点领域，全年计划实施主要建设项目416个，总投资3241亿元，年度计划完成投资1080亿元以上。重点推进金大地二期、卫龙产业园二期、宏途食品等134个工业项目；豫中农产品电商批发园、龙江现代物流园、万邦农产品冷链物流园等120个服务业项目；新西环、沙颍河复航工程、许信高速漯河段等90个城建交通项目；静脉产业园、污水处理厂等21个节能环保项目；市美术馆、中小学建设等51个社会事业项目，为高质量发展蓄积强大的后劲。

强化项目管理服务。健全月通报、季讲评季开工、半年观摩、全年奖惩推进机制，坚持重点项目建设周例会、项目台账管理等制度，强化项目协调、跟踪问效，确保项目真开工、快建成、早见效。推进分类分级决策许可，实施容缺受理、并联审批，加快项目建设规划、土地手续办理。深化城镇规划区建设土地、农村耕地、乡村集体建设用地综合改革，完善土地收储机制，切实保障重点项目建设用地，力争全年收储土地5000亩以上，盘活存量建设用地1000亩以上，报批土地8000亩以上。坚持依法征迁、阳光征迁、和谐征迁，实施征迁攻坚，破解征迁难题。用足用活中央增加地方政府专项债券政策，积极谋划争取上级政策性项目资金。加快预算内资金拨付，力争9月末项目资金拨付大头落地。深化与开发性金融机构合作，定期开展融资对接活动。

加强重大项目储备。在产业升级、交通能源、公共服务、生态环保、民生改善等领域，分近中远期谋划储备一批重大项目，形成项目储备和滚动接续机制，更好发挥有效投资对优化供给结构的关键性作用。抓实抓细重点项目库、百城提质和房地产项目库、“三大改造”项目库、招商引资履约项目库与统计数据库的联动对接，实现投资项目“五库联通”。

（二）持续优化供给结构，培育高质量发展的产业体系。深入推进供给侧结构性改革，优化产业空间布局，推进产业结构调整，加快构建产业新体系、培育发展新动力，增创经济高质量发展新优势。

加快推进工业转型升级。深入实施工业“三大改造”，全年完成投资213亿元，抓好207个重点改造项目，推广应用500台套以上机器人及智能装备、60个以上企业生产线智能化改造，推动300家以上工业企业上云。进一步提高食品产业集中度，努力打造完善丰满的食品产业生态，让“中国食品名城”的品牌越擦越亮。按照“延伸产业链、提升价值链、打造供应链”的要求，着力打通食品产业和装备制造业，培育食品机械产业；打通食品产业与造纸产业，发展食品包装产业；打通食品产业和医药产业，发展医用食品、功能性食品、保健性食品，积极引进生物医药产业；推动食品产业向宠物食品和动物饲料延伸；加快食品物流尤其是冷链物流、食品检验检测产业发展。强化食品安全、食品质量监管，加快食品标准化体系建设。完善食品共性技术研发中心、协同创新中心功能，支持重点食品企业创新平台、重点试验室建设。强化与知名高校、院所合作，支持漯河食品职业学院、漯河医专、漯河职业技术学院、漯河技师学院硬件建设和内涵提升，加快产学研融合和技能型人才培养。办好第十七届中国（漯河）食博会，进一步提高行业影响力。组建食品产业基金，引导食品企业集中集聚。全力支持双汇建设总部基地，支持南街村等重点食品企业新上项目，加快平平、旺旺、统一、大沛、美国嘉吉预混料、中大生物甜菜红等项目，支持经开区食品机械包装产业园、临颍包装材料产业园建设。积极发展壮大装备制造产业，重点围绕装配式建筑、液压科技、精密制造、电力装备等领域，抓好天成鸿路绿色建筑产业园、流体科技产业园、英利电子科技、永光电器设备等项目，着力打造全省绿色装配式建筑产业基地和全国有影响的流体科技产业基地。推动化工产业向价值链高端、产业链终端发展，提升循环化利用和绿色发展水平。以舞阳盐化工产业园、漯西化工园为载体，进一步补链、延链、强链，加快推进金大地改扩建、银鸽综合产业园等项目，大力发展精细化工、医药化工等精深加工产品。着力培育医药医疗器械、电子信息、新能源新材料、大数据等产业，支持曙光健士、乐通源德福、协鑫新能源等企业发展壮大，开工建设华为大数据产业园，引入产业链核心企业，努力培育新的增长点。强化载体建设，支持临颍产业集聚区升级为国家级经济技术开发区、舞阳产业集聚区升级为省级经济技术开发区，推动经开区和漯西化工园扩区，加大杜曲现代家居产业集聚区和沙澧产业集聚区高铁以西区域市场化试点推进力度。着力谋划建设专业园区，推动食品机械包装、流体科技、电子信息、电力装备等专业园区围绕细分领域链条式、集聚化发展，使之成为产业集聚区内的“专业店”、“精品店”。探索“管委会+公司”、“园区+创投”等市场化开发运营模式，加快产业聚集区提质增效。

加快发展现代服务业。全年实施服务业项目120个，总投资904亿元，年度完成投资300亿元，推动服务业扩量提质、结构优化、占比提升。着力发展冷链物流、快递物流、电商物流，建设全国冷链物流中心、全国快递示范城市、全省商贸物流标准化城市。加快推进传化物流、普洛斯仓储物流、临颍现代物流园等重点项目，抓好临港产业园申报国家级多式联运示范工程，着力打造豫中南现代物流核心区。大力发展电子商务，支持双汇、平平等骨干企业建立线上线下融合销售渠道，抓好春风吹来电商物流等项目建设，推动漯河电子商务产业园争创国家级电子商务产业示范园区。统筹推进6个服务业“两区”、4个省级服务业专业园区提质扩容，形成引领服务业高质量发展的重要支撑。市商务中心区和临颍、郾城特色商业区力争晋升二星级。积极发展新兴服务业，抓好科创产业园、市职教园区等园区建设，推进国家级肉制品检验检测中心市场化进程，加大金融机构和新型业态引进力度。推进健康、养老、育幼、体育、休闲、文化、信息等领域供给侧结构性改革，着力增强供给、提质产品、优化环境，满足群众中高端服务消费需求。落实个人所得税专项附加扣除政策、全面推行产品质量可追溯制度、严厉打击侵害消费者权益行为，让群众“能消费、愿消费、敢消费”。

加快发展现代农业。扛稳粮食安全重任，推动藏粮于地、藏粮于技，集中力量建设高标准粮田，统筹推进农技推广、农机装备、智慧气象等配套工程建设，稳步提升粮食产能。严守耕地保护红线，加强土地执法监管，坚决遏制“大棚房”、农地非农化和各类土地违法行为，保住粮食生产的命根子。深化农业供给侧结构性改革，加快推进“四优四化”，优质小麦种植面积稳定在100万亩以上；坚持“粮头食尾”“农头工尾”，加快农产品加工业转型升级，不断提高农业质量效益，实现粮食安全和现代高效农业相统一。突出抓好家庭农场、农民合作社、农业产业联合体等新型经营主体培育，强化金融支持，促进小农户和现代农业发展有机衔接。积极探索“农业+旅游”“农业+文化”“农业+保险”等发展模式，培育一批精品农业园区。推进农产品标准化生产，完善农产品原产地可追溯制度和质量标识制度，新认证“三品一标”农产品30个以上，力争全市80%以上生产经营主体及其产品实现可追溯，促进农业由总量扩张向质量提升转变。

（三）持续跟踪协调服务，壮大高质量发展的企业群体。经济形势越复杂，越要加大服务企业力度，推动资源要素向企业集聚、政策措施向企业倾斜、工作力量向企业加强，着力培育壮大一批支撑高质量发展的骨干企业群体。

大力实施重点企业倍增工程。全力以赴抓好去年以来谋划确定的双汇、银鸽、烟厂、际华3515五年倍增工程。今年重点支持双汇开工建设总部大厦，加快启动生产线智能化改造、第四方冷链物流平台和物流基地、快餐工程等一批重大项目建设；支持银鸽开工建设六基地填平补齐项目，推动包装纸、生活纸后加工产业园加快落地，启动漯西银鸽综合产业园建设；支持烟厂开工建设易地技改项目，力争三季度破土动工；支持际华3515建成投产工业园一期，全面启动二期项目。各县区、管委会也要结合实际，谋划实施重点企业倍增计划，强化财源支撑。

着力培育骨干企业。要像重视招商引资一样重视本土企业培育，持续深化“十百千”亿级企业及产业集群培育工程和“个转企”、“小升规”工程，着力培育上市企业、航母级新企业、小巨人级优势企业和细分领域的单项冠军企业，力争今年再入库规模以上工业企业50家以上，新增主营业务收入超亿元企业15家、超50亿元企业2家。持续提升服务企业周例会的精准度和实效性，着力解决工业运行、企业经营、项目推进中的困难和问题，集聚资金、科技、土地、人才以及政府资源等要素，在产业政策、项目审批、环保等方面给予有效支持。

全力支持实体经济发展。坚持问题导向，强化政策统筹协调，切实解决实体经济发展中的痛点难点问题。全面落实国家大规模减税和明显降低企业社保缴费政策，落实小微企业、科技型初创企业普惠性税收减免政策，全方位规范清理地方收费，专项治理中介服务收费，切实降低企业税费负担，让企业有实实在在的获得感。引导金融机构扩大信贷投放、降低贷款成本，精准有效支持实体经济。搭建政银企对接平台，让企业好项目和金融机构新产品开展路演，强化银企对接。研究建立政府出资引导、基金管理公司市场化规范运作的产业投资基金，支持企业转型、产业升级。完善上市激励政策，加大企业上市培育力度，争取更多企业实现上市直接融资。强化企业用工需求衔接，定期组织专场招聘活动。续建输变电工程11项，新开工输变电工程14项，不断提升供电保障能力。建立重点企业与政府的直通车机制，让企业的困难有处诉说、有人协调，及时帮助企业纾困解难。 （四）持续提升规划建设管理水平，构建高质量发展的城乡格局。以全国文明城市创建、百城建设提质暨城市“双修”为抓手，统筹推进新型城镇化和乡村振兴战略，加快形成城乡融合发展新格局。

着力推进城乡规划高质量。围绕“四城同建”，完善规划体系，持续推进城市“双修”和城市设计试点，加快形成城市设计体系，丰富城市内涵，塑造城市风貌。编制完善森林漯河、排水防涝、城市桥梁等专项规划和重点区域的详细城市设计，加快完成市区南部水系和湿地公园、示范性主干道路、示范性背街小巷等具体项目的规划和设计，形成“总体规划为统领、控制性详规为重点、专项规划为支撑、详细城市设计为基础”的全域覆盖、多规合一的规划体系。

着力推进城市建设高质量。全年计划实施中心城区百城提质项目299个，总投资500亿元，年度完成投资150亿元以上。新建改造淮河路、辽河西路等31条市区道路，打通交通北路、会展路等12条断头路，加快推进白云山路沙河桥、北舞渡沙河桥等16座桥（闸）工程。加快S222开遂线（西环）建设，谋划推动107国道至西环连接工程、南环路改造等重大交通项目。沙澧河大二期沙河沿线107国道至高铁段上半年完成景观建设、“六一”前开园迎宾，高铁以西到沙澧连通段年底前完成主体工程和景观绿化、明年初开园迎宾。沙澧连通工程年底前完成新河道开挖和节制闸、丹江路大桥建设，今冬明春完成景观绿化。幸福渠水系年底前实现高铁至交通北路段12.5公里全线贯通、开园迎宾。

西城区小南湖湿地公园和高铁以东水系、示范区沙河大二期淞江路至牡丹江路段景观工程“五一”前建成迎宾。示范区环形水系、龙江生态城水系要全面加快建设步伐。开工建设黑河综合治理工程，启动南部城区湿地水系前期工作。双龙区域11条道路的雨污分流工程要在今年汛期前全部完工。完成龙江路、中山路等14条道路的绿化提升，新建改造街头游园17个，建设生态绿廊20.7公顷。实施既有建筑温暖行动，新建供热管网30公里、换热站16座，新增供热面积138万平方米。新建雨污分流管网30公里以上。新建改造公厕35座。加快推进小李庄、五里庙等26个棚改项目。力争全面完成市区老旧小区、背街小巷整治。探索推进城市街区更新改造。进一步增强临颍、舞阳县城的承载能力和服务功能，加快推进县域经济高质量发展。结合区位条件、资源禀赋、产业优势，探索打造一批近郊特色小镇和乡村特色小镇。推行城建项目施工现场公开承诺制，向市民全面公示设计方案、责任单位、责任人、施工周期等信息，切实做到施工围挡少围一米是一米、少围一天是一天，以公开承诺赢得周边群众的理解和支持、体现建设单位的自信与担当。

着力推进城市管理高质量。强力实施城市“四治”，提高城市柔性化治理、精细化服务水平。结合机构改革，进一步理顺城市管理体制。加强智慧城市建设，提高“数字城管”执行效率。强化综合执法，探索“城管+”基层治理模式，推动执法事项属地化管理。开工建设生活垃圾焚烧发电、建筑垃圾消纳处理项目，启动餐厨垃圾处理项目前期工作。试点开展生活垃圾分类处理。严格落实“门前三包”责任制和网格化管理。深化经营秩序等专项整治。加强校园周边等重点部位和出租车等重点领域治理。完善智能交通管理系统，充分利用地下空间，加快停车场、停车站点等交通设施建设，多措并举解决交通拥堵和停车难问题。对22条道路人行道进行无障碍改造。为19个社区、120条小巷新装路灯。积极支持老旧小区加装电梯。着力提升社区物业服务水平，努力实现物业管理服务全覆盖。

着力推进城市经营高质量。突出城市土地、城市设施、城市环境三大重点，坚持以经营的理念规划城市、以经营的手段建设城市、以经营的方式管理城市，整合城市资源，盘活城市资产，探索市场主导的城市建设投资模式。严格用地管制，规范交易秩序，清理闲置土地。强化土地收储新机制，做到土地收储与基础设施同步谋划、同步征迁、同步实施。

着力推进乡村振兴高质量。认真学习贯彻习近平总书记在参加十三届全国人大二次会议河南代表团审议时的重要讲话精神，加快推进产业、人才、文化、生态、组织“五个振兴”。筹措不少于1亿元统筹用于乡村振兴和“三农”工作。按照先规划后建设的原则，加快编制多规合一的实用性村庄规划。借鉴浙江“千村示范、万村整治”经验，发动农民参与，注重实际效果，扎实推进农村人居环境整治。完善农村保洁市场化运作机制，做好农村生活垃圾、污水治理工作。积极推进“厕所革命”，改造农村户厕9.8万户。大力开展“四美乡村”创建，力争创建示范乡镇10个、示范村100个、示范户2万户。补齐农村基础设施短板，重点抓好农村交通运输、农田水利、农村饮水、乡村物流、宽带网络、电网改造等建设，逐步建立全域覆盖、普惠共享、城乡一体的基础设施服务网络。加强农业生态环境保护和农村污染防治，完善农产品产地环境监测网络，保护好水源和土壤，净化农产品产地环境。夯实乡村治理根基，健全村级议事协商制度，推进村级事务公开，加强村级权力有效监督。推进移风易俗，整治农村婚丧大操大办、高额彩礼、铺张浪费、厚葬薄养等不良习俗，培育文明乡风、良好家风、淳朴民风。

（五）持续深化改革开放创新，筑牢高质量发展的动力系统。坚持以深化改革激发动力，以扩大开放焕发活力，以矢志创新迸发潜力，筑牢高质量发展的动力系统。

推进重点领域改革。深化国企国资改革，全面完成省定7家“僵尸企业”处置后续工作。加快推进市属国有非工企业改革。加快市城建投公司、市发投公司等国有投融资平台改革，建立健全现代企业制度。改革国有资本投资运营体制，强化国有资产基础管理，推进经营性国有资产集中统一监管。加快农信社改革，大力推进农商行组建。持续深化农村改革，深入推进农村集体产权制度改革，建立健全农村集体资产管理制度，完善农村集体产权权能，发展壮大新型集体经济。稳妥推进农村土地“三权”分置改革，完善市、县、乡、村四级土地经营权流转服务体系。深化市、县机构改革，优化职能配置，提高行政效能。统筹推进财税体制、盐业体制、医药、教育等其他领域改革。

全面推进对外开放。按照产业和招商图谱，深化与央企、行业龙头企业战略合作，坚持大员上阵，实施精准招商，力争全年新签约超亿元项目100个以上，超10亿元项目20个以上。大力实施出口品牌战略，培育出口企业的国际竞争力。全力服务双汇、际华3515等进出口大户和利通公司、南街村等有望扩大进出口规模的企业。加快开放平台建设，积极参与“一带一路”建设，深化与郑州航空港战略合作。加快海关业务用房和监管场所建设，推动漯河海关早日开关运行。积极推进漯河公共保税区建设。充分发挥进口肉类指定口岸、跨境电商示范园等平台作用，吸引企业来漯开展业务。

抓好创新驱动发展。启动市级科技创新重大专项，争取列入省“十百千”转型升级创新专项。推进实施创新引领型企业、人才、平台、机构等“四个一批”行动计划，实现全面提质增效。强化企业孵化器和“双创”平台打造，加快数字化利通橡塑创新研究院等新型研发机构建设。深化与中科院的战略合作，鼓励企业联合高校、科研院所，建立技术研发中心、工程技术中心和重点实验室。深入实施创新型龙头企业培育工程，支持开展技术和商业模式创新，新培育国家级高新技术企业10家、市级15家以上。加大研究与试验发展经费投入强度，加快科技成果转化速度。落实人才激励政策，做好人才引进工作。继续推行科技创新券制度，支持企业科技创新。实施标准化发展战略，开展食品安全、商贸物流标准化专项行动，争创国家标准化综合改革试点市。

（六）持续打好三大攻坚战，补齐高质量发展的短板弱项。把打好三大攻坚战摆在全局更加突出的位置，精准发力，全力攻坚，务求实效。

打好防范化解重大风险攻坚战。加强金融监管，持续开展非法集资、P2P网络借贷等专项整治。强化地方政府债务全方位监管，坚决制止违法违规和变相举债行为，积极稳妥化解存量债务。开展投资类企业风险排查和分类监管。加强企业互保行为监管，防范企业担保链风险。抓好食品药品安全监管，建立食品安全追溯系统，健全食品安全应急处置机制，争创全国食品安全示范市。整合检验检测资源，组建高标准的食品安全检验检测机构。强化重大动物疫病防控，保持非洲猪瘟防控“零疫情”。完善安全生产风险隐患双重预防体系，不间断地排查、整改、消除安全隐患，加强重点领域专项整治，坚决防范遏制重特大事故发生。加快完善应急管理体系，做好突发公共事件预防处置。及时化解各类信访矛盾，防范社会稳定风险。

打好精准脱贫攻坚战。坚持精准方略，着力解决好“两不愁三保障”面临的突出问题，加大特殊贫困群体脱贫攻坚力度，减少和防止贫困人口返贫，抓好贫困“边缘户”帮扶，确保完成省定脱贫目标任务。把产业扶贫作为脱贫攻坚的根本措施，重点支持贫困村、贫困户因地制宜发展种养业和加工业，多渠道开发适合贫困劳动力的就业岗位，实现长期稳定脱贫。严格落实医保救助政策，加强健康扶贫“一站式”结算机制建设，扎实开展21种大病集中救治、慢性病诊疗和签约医生服务，切实减轻贫困群众的看病负担。引导贫困群众自立自强，消除“等靠要”思想，培育脱贫致富光荣的良好社会风尚。

打好污染防治攻坚战。坚守环境质量只能变好、不能变坏这一刚性底线。打好蓝天保卫战，推进煤炭消费减量、产业布局优化、运输结构调整、生态扩容提速、柴油货车治理、扬尘治理提效、工业绿色升级、清洁取暖推进、监测能力提升、秋冬污染防治等十项攻坚，聚焦控尘、控排、控煤、控烧、控车、控油和重污染天气应急应对等“六控一应对”，突出抓好重点区域、重点时段、重点行业、重点因子等“四个重点”。全面推进森林漯河建设，大力实施生态林业重点工程，完成营造林7万亩以上。打好碧水保卫战，实施“四水同治”。完善河长制长效机制。以饮用水源保护、河流断面达标和黑臭水体治理为重点，加快澧河取水口上移、淞江产业集聚区生态水系修复、黑河西支水体整治等工程建设，推进召陵东城污水处理厂三期扩建、马沟污水处理厂二期和沙澧产业集聚区污水处理厂二期等5个污水处理项目，完成现有18个县级以上污水处理厂提标改造。打好净土保卫战，开展农用地土壤环境质量类别划分，完成省定农用地安全利用、治理与修复、种植结构调整任务，严格污染地块准入管理，抓好农业面源污染防治和固体废物处理，加强污染地块风险管控和治理修复，重金属污染物排放量实现零增长，土壤污染防治体系逐步完善，土壤环境风险得到基本控制。

（七）持续完善体制机制，打造高质量发展的营商环境。打好优化发展环境“组合拳”，营造便利高效政务环境、开放公平市场环境、公正透明法治环境、诚实守信信用环境，努力打造全国、全省营商环境新高地。

持续深化“放管服”改革。纵深推进“一网通办”前提下“最多跑一次”改革。加快数字政府建设。完善一体化在线政务服务平台。大力推进审批服务“三集中、三到位”，除对场地有特殊要求的事项外，审批服务事项进驻综合性实体政务大厅应进必进，90%以上审批服务事项“一窗”分类受理。推动企业和群众办理审批服务事项“马上办、网上办、就近办、一次办”，努力实现行政审批事项全省最少、审批流程全省最优、服务效率全省最高的目标。推行“证照分离”改革，克服企业“准入不准营”现象。加快推进信息共享集成、流程集成、人员集成步伐，进一步缩短不动产登记办理时限。深化投资项目审批制度改革，力争38天内完成企业投资项目全流程审批。加快推进工程建设项目审批制度专项改革。推行“区域评估”多评合一，实现区域内建设项目共享共用成果，通过政府买单、企业共享，加快项目落地。健全统一的“12345”政府服务热线，实施一号对外、多线联动，全天候受理，解答、转办群众和企业咨询投诉建议。

深化商事制度改革。推进经营场所登记制度改革，允许创办企业注册登记实行“一照多址”和“一址多照”。建成“互联网+监管”平台，推进“双随机、一公开”，实现跨部门联合监管。推动社会信用体系与大数据融合发展，完善跨区域、跨行业的信用信息共享机制。推行信用监管改革，建设全市一体化的公共信用信息共享平台，建立常态化的诚信“红黑名单”定期发布机制。全面推行守信联合激励、失信联合惩戒，健全以信用为核心的新型市场监管机制，争创国家社会信用体系建设示范市。

营造亲商富商的良好氛围。坚持“两个毫不动摇”，落实民间投资支持政策，更加重视、支持民营企业发展，着力构建亲清新型政商关系。大力弘扬企业家精神，宣传优秀企业家典型事迹，激发企业家创新活力和创造潜能。切实保护民营企业家的人身安全和财产安全，让民营企业家放手发展、放心发展。启动营商环境评价，实施营商环境核心指标对标优化行动。加强部门协同，打通政策落实、项目建设“最后一公里”。继续将周例会作为服务企业发展、推进项目建设的重要平台，畅通社会各界提交周例会议题的渠道。进一步提升周例会工作实效和质量，对周例会议定事项定期督导、定期通报，凡是该解决的问题不解决不下周例会。

（八）持续增进人民群众福祉，夯实高质量发展的社会基础。坚持以人民为中心的发展思想，尽力而为、量力而行，完善制度、守住底线，集中力量办好一批重点民生实事，实现经济发展与民生福祉互促共进。

提高就业质量。把稳就业放在更加突出位置，做好高校毕业生、退役军人、城镇困难人员、农村转移劳动力等重点群体就业工作，支持外出务工人员返乡就业创业。大力推进以创业带动就业，积极推进公共就业服务专项活动，持续实施全民技能振兴工程，推动实现更高质量和更充分就业。

加快公共服务体系建设。努力办好人民满意的教育。今年要围绕解决城镇中小学“大班额”问题，重点抓好投资22亿元的20所城镇中小学建设，其中中心城区新建10所、续建4所、扩建2所，临颍、舞阳两县共新建2所、续建1所、扩建1所。这20所中小学建设要全部实施台账管理，由市政府挂牌督办，彻底补齐中心城区尤其是沙北区域中小学学位供给严重不足的短板。继续实施第三期学前教育三年行动计划，新建、改扩建幼儿园58所。调整农村中小学布局，改扩建农村寄宿式学校23所。大力发展职业教育，加快培养产业发展急需的技术能手、技能人才，让更多的漯河青年凭借一技之长实现人生价值。抓住国家扩大高职院校招生规模的机遇，支持漯河医专、漯河职业技术学院、漯河技师学院、漯河食品职业学院扩大对外合作，推进产教融合、校企合作和实训基地建设。全力支持漯河食品职业学院完善升本的各项条件。加快推进漯河技师学院新校区、市一中专新校区等项目建设。持续推进区域医疗中心建设，建成投用市中心医院全科医师培训基地。加快推进市二院儿童医疗中心、召陵区中医院综合病房楼建设。实施临颍、舞阳县级医院提升工程。深化公立医院综合改革。完善公共卫生服务体系。围绕打造中华汉字文化名城，深入挖掘和展示汉字文化。挖掘保护“沙河调”、传承推广“心意六合拳”等地域传统特色文化。广泛开展全民健身活动，积极举办各类体育赛事。引导和支持志愿者服务体系建设。建成投用市科技馆，加快市示范性综合实践基地、全民健身活动中心、舞阳贾湖遗址保护等项目建设。加快市殡仪馆易地建设。推进市养老产业综合服务园、市医养产业园、舞阳和郾城老年养护楼等项目建设。

完善社会保障体系。完善城乡居民医保门诊统筹制度，推动城乡居民基本医保与大病保险制度有效衔接、生育保险和职工基本医疗保险合并实施。提高基本医保、大病保险、困难群众大病补充医疗保险保障水平。提高城乡居民基本养老金最低标准，调增企业和机关事业单位退休人员基本养老金。提高特困供养、孤儿养育保障标准。加强残疾人权益保障，对残疾儿童实施康复救助。继续免费开展预防出生缺陷产前筛查、新生儿疾病筛查和“两癌”筛查。新建500个“儿童之家”。提高对城乡低保对象等特殊群体的政策性补贴。从1月1日起向全市80岁以上老人发放高龄津贴。建成市、县、乡、村退役军人四级服务保障体系，落实退役军人待遇保障，完善退役军人基本养老、基本医疗保险接续政策。加快建立多主体供给、多渠道保障、租购并举的住房制度，新开工棚改安置房3000套、基本建成1500套以上。

加强和创新社会治理。学习推广“枫桥经验”，高质量推进“一村（格）一警”工作，全面加强基层社会治理。健全救援工作机制，提高防灾减灾救灾能力。完善公共法律服务体系。完善预防和化解社会矛盾机制，加强信访法治化建设。健全立体化信息化社会治安防控体系，深入推进扫黑除恶专项斗争。扎实做好庆祝建国70周年相关工作。

加强国防教育、国防动员和国防后备力量建设，支持驻漯解放军、武警部队、预备役部队建设，抓好民兵和人民防空建设，深入开展双拥共建，深度推进军民融合发展，巩固军政军民团结。全面贯彻党的民族政策和宗教工作基本方针，加强基层基础建设，促进民族团结、宗教和睦。加强统计工作，搞好第四次经济普查。支持工会、共青团、妇联更好发挥桥梁纽带作用。做好外事、侨务、港澳、对台工作。抓好参事、气象、地震、测绘、地质、邮政等工作。

各位代表！全面推进“四城同建”是市委七届七次全会作出的战略部署，市委七届九次全会进行了全面安排。我们要把“四城同建”作为全市经济社会发展的重中之重，凝心聚力抓实抓好。要深入落实1个指导意见、4个实施意见和4个年度工作方案，抓紧制定配套政策措施，不断完善“1+4+4+N”体系；充实“四城同建”项目库，与文明创建暨百城提质、城市“双修”重点项目库等有机融合；建立健全日常推进、台账管理、配合联动、督导考核等工作机制，抓好工程性项目建设、非工程性工作任务和招商引资，带动各项重点任务落到实处，确保“四城同建”年度目标圆满完成。

三、加强政府自身建设

进入新时代，政府工作要有新气象、新担当、新作为。我们要更加注重加强自身建设，着力提升治理能力，全面增强公信力和执行力，努力打造人民满意的政府。

（一）坚定政治信仰。深切领悟习近平新时代中国特色社会主义思想，始终树牢“四个意识”，坚定“四个自信”，坚决做到“两个维护”，自觉在政治立场、政治方向、政治原则、政治道路上同以习近平同志为核心的党中央保持高度一致，坚决贯彻落实党中央国务院大政方针和省委省政府、市委各项决策部署，确保政令畅通、令行禁止。坚决按照全面从严治党“八个坚定不移”的要求，落实主体责任，严明政治纪律和政治规矩，营造风清气正的政治生态。

（二）坚持依法行政。严格遵守宪法和法律，坚持依法施政、依法行政、依法办事，善于运用法治思维和法治方式开展工作、解决问题、推动发展。严格执行民主集中制，坚持科学、民主、依法决策。探索建立“开门决策”机制，拓展公众参与重大行政决策渠道。加强行政执法监督和责任追究，规范文明执法。完善府院联动和“两法衔接”机制，依法解决历史遗留问题和行政复议执行难等问题。建设契约政府、诚信政府，持续纠正“新官不理旧账”的问题。深入推进“七五”普法。自觉接受人大法律监督、工作监督和政协民主监督、专门机关监督，强化审计监督，主动接受社会和舆论监督，广泛听取人大代表、政协委员、民主党派、工商联、无党派人士和各人民团体的意见，高质量办好人大代表建议和政协提案。深入推进“阳光漯河”建设，全面推进政务公开，让权力在阳光下运行。

（三）坚决扛起担当。学习弘扬焦裕禄同志对群众的亲劲、抓工作的韧劲和干事业的拼劲，面对矛盾敢于迎难而上，面对危机敢于挺身而出，面对失误敢于承担责任，真正把担当作为行动自觉和职业习惯。切实增强“八种本领”，做到“五个过硬”，坚决克服思想懈怠、不思进取、本领恐慌，努力成为各领域敢于担当的行家里手。持续提升政府执行力，善于在推动高质量发展中研究政策、发现机遇、破解难题，探索推行清单化、项目化管理，实施执行落实效果动态评估，全面提升行政效能。落实“三个区分开来”，健全正向激励、容错纠错、澄清关怀三项机制，明确权责清单，保护改革者，鼓励探索者，宽容失误者，让广大干部消除顾虑、卸下包袱，甩开膀子干事创业、建功立业。

（四）坚守为民宗旨。始终把人民放在心中最高位置，既要谋划实施好普惠性的民生大事，更要用心办好群众家门口的“暖心”实事，千方百计让人民群众生活得更安心、更省心、更舒心。严控“三公”经费预算，压减一般性支出5%以上。全面实施预算绩效管理，把有限财力用在惠民生、干实事上。深入纠“四风”、持续转作风，统筹规范督查检查考核事项，坚决反对一切形式主义、官僚主义，让干部从文山会海、迎评迎检、材料报表中解脱出来，把更多精力用在解难题、谋发展上。坚定不移反腐倡廉，严格落实中央八项规定、实施细则以及省委、市委相关规定，认真对待群众反映的问题，坚决整治损害群众利益的不正之风和腐败行为。

各位代表！使命重在担当，实干成就未来。让我们紧密团结在以习近平同志为核心的党中央周围，高举中国特色社会主义伟大旗帜，在省委省政府和市委的坚强领导下，团结拼搏、锐意进取，深入落实新时代漯河经济社会发展“四三二一”工作布局，全面推进“四城同建”，努力开创高质量发展新局面，以优异成绩迎接中华人民共和国成立70周年！
